# Implementation of Dietary Reference Intake Standards in Prison Menus in Poland

**DOI:** 10.3390/nu12030728

**Published:** 2020-03-10

**Authors:** Piotr Stanikowski, Monika Michalak-Majewska, Dorota Domagała, Ewa Jabłońska-Ryś, Aneta Sławińska

**Affiliations:** 1 Department of Plant Food Technology and Gastronomy, Faculty of Food Science and Biotechnology, University of Life Sciences in Lublin, 20-704 Lublin, Poland; monika.michalak@up.lublin.pl (M.M.-M.); ewa.jablonska-rys@up.lublin.pl (E.J.-R.); aneta.slawinska@up.lublin.pl (A.S.); 2 Department of Applied Mathematics and Computer Science, Faculty of Production Engineering, University of Life Sciences in Lublin, 20-612 Lublin, Poland; dorota.domagala@up.lublin.pl

**Keywords:** dietary reference intake, prison diets, nutrients, prisons, detention centers, recommended dietary allowance

## Abstract

Adequate nutrition in prisons should constantly be monitored due to the limited possibilities of external control as well as the low catering budget for prison meals and poorly defined requirements in this regard. The aim of the study was to assess the nutritional value of meals served in Polish prisons. Using a computer program, 14-day regular and bland diets from 30 prisons were analyzed. The energy value of the meals and the percentage of energy provided by protein, fat, and carbohydrate contained therein were found to meet the recommendations of the Polish National Food and Nutrition Institute. The amount of minerals supplied with the diet did not cover the recommended dietary allowance (RDA) in the case of calcium and magnesium. Particularly disturbing was the excessive supply of sodium in the regular and bland diets, which covered 537% and 311% of the dietary reference intake (DRI), respectively, as well as phosphorus (194 and 192% of RDA). The largest vitamin deficiencies were recorded for vitamins D and C and folate. An especially excessive supply was observed for vitamins A and B_12_. The type of diet significantly differentiated the average content of over half of the analyzed components, whereas the season of the year turned out to be statistically insignificant. The results of the present investigations indicate a need for development of more accurate legal provisions to regulate the nutrition in Polish prisons in terms of not only the energy value and macronutrient supply but also the intake of minerals and vitamins.

## 1. Introduction

In Poland, there are currently 130 independent prisons with a total of 74,696 inmates (data from 08.12.2019) [1].

One of the basic rights granted to imprisoned subjects in Poland is the right to adequate nutrition corresponding to the health status [2]. All relevant issues are regulated by the Regulation of the Minister of Justice of February 19, 2016. Polish prisons serve regular diet, diets for juveniles under 18 years of age, therapeutic diets (bland, diabetic, individual-adjusted), extra meals for prisoners working in arduous conditions, and meals adjusted to religious and cultural requirements. Each meal is complemented with a beverage, i.e., potable water, tea, or cereal coffee. The energy value of meals should not be lower than 2600 kcal or 2800 kcal in the case of detained juveniles, and 10–15% of energy should come from protein, less than 30% from fat, and 50–65% from carbohydrate. The quantitative content of vegetables and fruits (not including potatoes) should be at least 300 g per day [3].

A healthy balanced diet containing plenty of fruit and vegetables, low in fat, salt, and sugar, and based on whole-grain products is an important determinant of prisoner health [4]. However, the prison meals, the possibility to buy additional food in canteens, and limited physical activity may exert negative effects. Weight gain has been noted in 42–75% of male convicts during imprisonment [5]. A nutrient-poor diet may promote antisocial behaviors in prisoners as well [6]. Prisoners are significantly more frequently burdened with psychiatric and physical disorders than the general population. This is caused by many risk factors that affect especially this group; these mainly include drug use, alcohol misuse, and tobacco smoking [7]. These behaviors contribute to an increased risk of cardiovascular disease in these subjects [8]. In comparison with the general population, prisoners more often suffer from hypertension, asthma, arthritis, and hepatitis [9]. In Canada, 9% and 5% of prisoners were diagnosed with hypertension and with high cholesterol levels, respectively, whereas diabetes affected 4% of inmates [10]. Special diet following nutritionist recommendations may significantly reduce body weight, fat tissue, and diastolic blood pressure in prisoners [8].

Assessment of the dietary supply of selected nutrients in compliance with nutritional requirements has been conducted in only a few Polish prisons to date. Based on menus from five prisons, Kucharska et al. [11] showed excessive supply of sodium, potassium, phosphorus, and iron as well as vitamins A, E, B_6_, and B_12_; excessive energy value of meals was reported from two of these prisons. Additionally, calcium, copper, and vitamins B_2_, C, and D deficiencies were detected. Another study carried out in the Warsaw-Grochów Detention Center revealed excessive energy amounts and a deficient level of dietary fiber in diets for women relative to the nutritional needs in this group of inmates. The analysis of the content of vitamins and minerals demonstrated a higher level of vitamins A, E, B_2_, and B_12_ as well as sodium, phosphorus, and zinc than the recommended values. The daily supply of vitamins D, C, folate, potassium, calcium, and magnesium was insufficient [12]. 

The aim of the present study was to assess the nutritional value of meals served in some Polish prisons, and to compare the obtained values to the dietary reference intake standards.

## 2. Materials and Methods

### 2.1. General Information 

The study was approved by the Director-General of the Prison Service in Poland on December 3, 2018 and by 10 of the 15 Regional Inspectorates. In total, 30 independent prisons, i.e., 37% of the total number of penitentiary institutions, were selected for the investigations. Three prisons from each of the 10 Regional Inspectorates of the Prison Service located in central and eastern Poland were randomly selected, i.e., detention centers: Elbląg, Grójec, Gdańsk, Kraków, Łódź, Mysłowice, Sosnowiec, and Warsaw-Grochów; prisons: Barczewo, Biała Podlaska, Białystok, Bydgoszcz-Fordon, Chełm, Czerwony Bór, Dębica, Dublin, Garbalin, Grądy Woniecko, Grudziądz (No. 1), Iława, Inowrocław, Malbork, Nowy Sącz, Przemyśl, Rzeszów, Siedlce, Sieradz, Wadowice, Wojkowice, and Zamość. Most of the institutions were male prisons, whereas six facilities were female and male prisons. All prisons were for adults [13]. Next, a request for access to the menus was sent in an electronic or paper form to all the institutions. Another request for information about whether the prisoners consumed entire meals (basis of daily observation) and whether there were complaints about the quality of nutrition (basis of official statistics) was sent to supervisors of meal delivery.

### 2.2. Analysis of Energy and Nutrient Content

14-day regular diet menus (7 days from the spring–summer season and 7 days from the autumn-winter season) and 14-day bland diet menus (7 days from the spring-summer season and 7 days from the autumn-winter season) provided by each prison in 2018 were analyzed. The regular diet was served to all healthy adult prisoners, while the bland diet was prescribed by medical staff only temporarily [3]. In the study, 840 all-day menus consisting of breakfast, lunch, and dinner were analyzed. A typical breakfast usually consisted of wheat bread, margarine, and sandwich meats. Various types of soup and a dish composed of meat, potatoes, and side salad were served for dinner. Dinner mostly included wheat bread, margarine, and sandwich meats/cottage cheese/fish paste. With each meal, prisoners made cereal coffee (coffee substitute) or tea themselves. The calculations did not include food that prisoners were able to buy at least three times a month in the prison canteen or food parcels that prisoners received once a month from their relatives [2]. 

The quantitative analysis was carried out with the use of specialized software Dieta 5d (National Food and Nutrition Institute, Warsaw, Poland), which mainly incorporates Polish Food Composition databases developed at the National Food and Nutrition Institute in Warsaw [14,15] and the database of the United States Department of Agriculture [16]. All food products specified in the menus and inventory reports were analyzed. Inventory reports included names of the food products and their quantity in kilograms/liters used in the kitchen to prepare all meals. Ready meals included in the software database were not taken into account in the analysis. When a food product was not found in the software, its substitute was used (in accordance with the software instructions). The study involved assessment of 42 parameters of daily food rations: energy value, energy supply (from protein, carbohydrate, and fat), total protein, total fat, total carbohydrate, dietary fiber, amino acids (isoleucine, leucine, lysine, methionine, cysteine, phenylalanine, tryptophan), sucrose, cholesterol, fatty acids (saturated, monounsaturated, polyunsaturated, α-linolenic, linoleic), minerals (sodium, potassium, calcium, phosphorus, magnesium, iron, zinc, copper, manganese), and vitamins A (as retinol activity equivalents), retinol, B_1_, B_2_, niacin, B_6_, B_12_, C, D, E, and folate (as dietary folate equivalents). The results took into account averaged technological losses caused by heat treatment suggested by the manufacturer of the menu analysis software: folate and vitamin C–50%, vitamin B_1_–30%, vitamin B_6_–25%, vitamins A, E, retinol, and niacin–20%, and other parameters–10%. Next, the percentage of the daily reference intake was calculated based on nutrition standards for the Polish population [17]. Since approximately 57% of male prisoners in Poland in 2018 were in the age range of 31–50, the calculations were based on recommendations for this group [18]. For calculation of energy requirement, the physical activity level (PAL) of 1.4 was adopted.

### 2.3. Statistical Analysis

The statistical analysis, carried out using the Statistica 13.1 program (StatSoft, Cracow, Poland), was based on the average content of nutrients in the menus from each prison. A two-factor analysis of variance was used to check whether the type of diet and season affect the content of the analyzed nutrients. In the next step, 95% confidence intervals for differences between the means of 24 significantly differing components were determined. Principal component analysis (PCA) was used to present their structure. The next step consisted in hierarchical cluster analysis (HCA), performed for better presentation of the data structure. 

Grouping was carried out with the Ward method, using a distance matrix based on the Euclidean distance. Due to their decreasing similarity, the results were presented in a dendrogram illustrating the structure of the data set.

## 3. Results

### 3.1. General Characteristics

The mean all-day purchase-only cost of prison-provided meals and beverages was 4.22 ± 0.25 PLN (approximately EUR 0.99) for the regular diet and 5.19 ± 0.15 PLN (approximately EUR 1.22) for the bland diet. Inmates in 24 of the analyzed prisons complained about the quality of nutrition. Most often, they reported small portions, low energy value of meals, and low quality of sandwich meats and dishes. According to the information provided by prison employees, the amount of food consumed by the prisoners from their meals was 91% ± 8%. 

### 3.2. Energy and Macronutrients

Table 1 shows the results of the average energy value and macronutrients content in the meals served in prisons. The average energy value of the regular and bland diets was 2575.5 kcal/day and 2569.5 kcal/day, respectively. These values met the recommendations of dietary energy intake for the Polish population. However, they did not meet the recommendations specified by the Regulation of the Minister of Justice for Polish prisons [3]. The proportion of energy provided by protein, fat, and carbohydrate was 13.6%, 29.3%, and 57.1% in the case of the regular diet and 14.7%, 28.1%, and 57.1% in the bland diet, which was within the recommended range. The structure of fats consumed in both diets did not differ from the recommendations. Only the content of saturated fatty acids exceeded the recommended supply. The cholesterol content was 234.7 mg in the regular diet meals and 197.0 mg in the bland meals. The daily fiber supply constituted 126% and 111% of the adequate intake (AI), respectively. 

### 3.3. Minerals

Table 2 presents the results of the average supply of selected minerals in the prison menus. The recommended intake was most highly exceeded in the case of sodium. Its supply in the regular diet was 8048.3 mg, which accounted for 537% AI. In the bland diet, it was supplied at a level of 4658.6 mg, i.e., 311% of the recommended value. Similarly, the recommended daily intake was exceeded in the case of manganese, phosphorus, copper, and iron. The supply of potassium, zinc, and magnesium was close to the demand for these elements. The lowest intake relative to the recommendations was reported for calcium. Its supply in the regular diet was 421.2 mg, which represented 42% of the recommended value. The bland diet provided 391.1 mg of the component, i.e., 39% of the recommended intake.

### 3.4. Vitamins

Table 3 presents the results of the average supply of selected vitamins in the prison menus. The dietary reference intake (DRI) value was exceeded to the greatest extent in the case of the vitamin A supply. The regular and bland diets provided 2222.3 mg (247% of the recommended value) and 2028.0 mg (225% of the recommended value) of this nutrient, respectively. The daily intake of three vitamins (D, C, and folate) was found to be insufficient. Both diets contained on average 5.1 µg of vitamin D, which covered 34% of the demand. The average content of vitamin C in the analyzed regular and bland menus was 57.5 and 51.9 mg, respectively, accounting for 64% and 58% of the recommended doses. In turn, the daily supply of folates in the analyzed menus was 223.5 and 216.2 mg, respectively, i.e., 56% and 54% of the recommended intake of the nutrient. 

### 3.5. Analysis of the Menus by Types of Diet and Season 

The type of diet had a significant effect on the differences in the average content of more than half of the analyzed nutrients, whereas the season turned out to be a statistically insignificant factor. The content of 10 food components, i.e., protein, isoleucine, leucine, lysine, methionine, cysteine, phenylalanine, tryptophan, niacin, and protein (% energy) was lower in the regular than bland diets (Table 4). The average content of the other 14 nutrients: fat, sodium, magnesium, iron, zinc, copper, manganese, retinol, vitamin C, vitamin B_12_, dietary fiber, cholesterol, total monounsaturated fatty acids, and fat (% of energy) was higher in the regular diet. At the 95% confidence level, the difference between the mean total protein content in the regular and bland diets was −7.14 g; i.e., it was not lower than −9.96 g and not higher than −4.32 g. The lower total protein supply in the regular diet resulted in a lower supply of exogenous amino acids. The difference between the average fat content in these diets amounted to 4.27 and was not lower than 0.34 g and not higher than 8.19 g. A particularly large difference was shown in the case of sodium, as its average intake with the regular diet was by 3389.71 mg higher than that in the bland diet. This had an impact on the value of DRI coverage, which was 537% and 311%, respectively. The most noticeable difference in the vitamin content was observed for vitamin B_12_. Its level in the regular diet was on average 3.55 µg higher than in the bland diet. 

**Figure 1:**
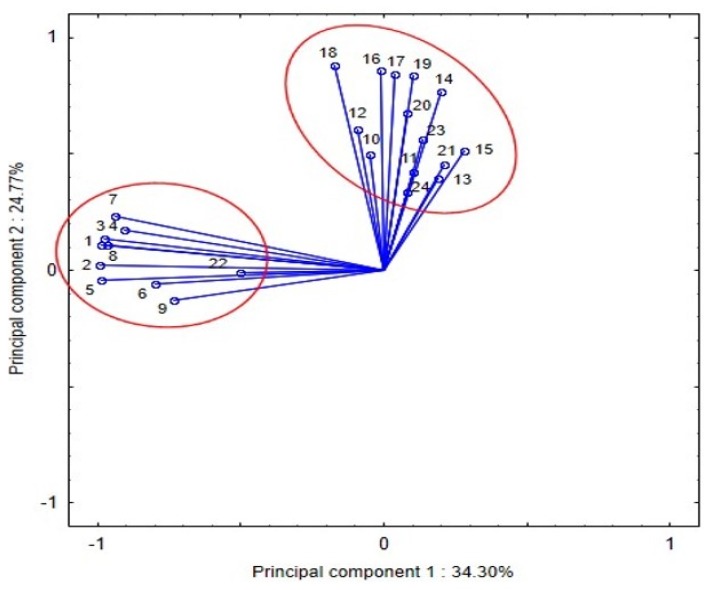
Projection of the components on the principal components plane (1—protein, 2—isoleucine, 3—leucine, 4—lysine, 5—methionine, 6—cysteine, 7—phenylalanine, 8—tryptophan, 9—protein (% of energy), 10—fat, 11—MUFA, 12—cholesterol, 13—fat (% of energy), 14—fiber, 15—sodium, 16—magnesium, 17—iron, 18—zinc, 19—copper, 20—manganese, 21—retinol, 22—niacin, 23—vitamin B_12_, 24—vitamin C).

PCA was used to represent the structure of the 24 essential nutrients. Figure 1 shows the projection of the components onto the plane determined by the first two principal components explaining 59.07% of the variance. It is evident that the food components form two groups. The first group comprises total protein, isoleucine, leucine, lysine, methionine, cystine, phenylalanine, tryptophan, protein (% energy), and niacin. The components in this group are most positively correlated at a level of 0.35–0.99. The second group contains the other nutrients, i.e., fat, sodium, magnesium, iron, zinc, copper, manganese, retinol, vitamin C, vitamin B_12_, dietary fiber, cholesterol, total monounsaturated fatty acids, and fat (% energy). The correlation between the components in this group is also positive but at a slightly lower level of 0.03–0.89. The correlation between the ingredients from the different groups is both positive and negative but substantially weaker. The highest Pearson correlation coefficient was calculated for protein (% energy) and monounsaturated fatty acids (−0.418) as well as protein (% energy) and sodium (−0.374). The lowest value (0.00002) was obtained for phenylalanine and manganese. 

It can be noted that the first group includes nutrients with lower average content in the regular diet. The division into these two groups coincides with the previous division into two groups of components whose average content is higher or lower in the regular and bland diets.

Next, the data structure was presented using cluster analysis. In the first step, the number of components was reduced. To this end, the results of the principal component analysis of the nutrients were used, and the components were grouped using cluster analysis. Taking into account the results of both analyses, six groups of nutrients were distinguished, two of which had only one component. Next, six variables representing each group were selected. Sodium and vitamin C represented single-element groups. The components in the other four groups were subjected to PCA, and the first main component of PCA was the representative of each group. The data were grouped based on variables defined in this way.

**Figure 2:**
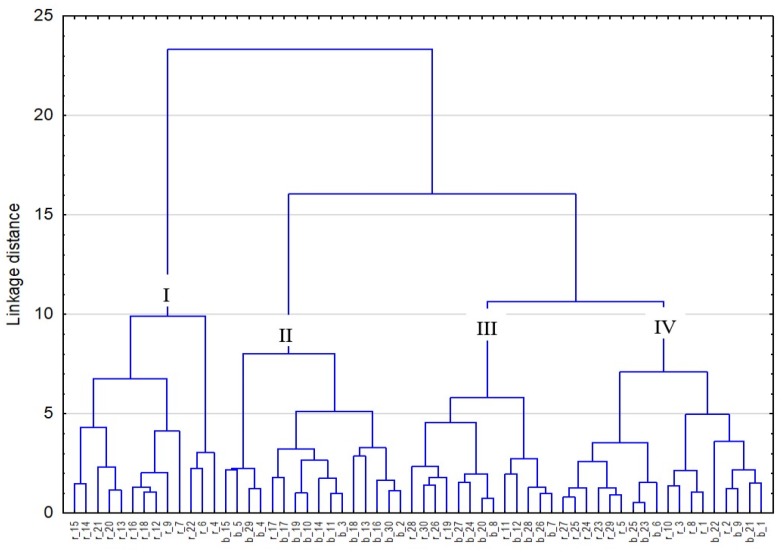
Dendrogram showing the results of the hierarchical cluster analysis (r—regular diet, b—bland diet, 1—Biała Podlaska, 2—Chełm, 3—Zamość, 4—Rzeszów, 5—Dębica, 6—Przemyśl, 7—Czerwony Bór, 8—Białystok, 9—Grądy Woniecko, 10—Grójec, 11—Siedlce, 12—Warszawa Grochów, 13—Nowy Sącz, 14—Kraków, 15—Wadowice, 16—Łódź, 17—Sieradz, 18—Garbalin, 19—Bydgoszcz Fordoń, 20—Innowrocław, 21—Grudziądz I, 22—Barczewo, 23—Dubliny, 24—Działdowo, 25—Elbląg, 26—Malbork, 27—Gdańsk, 28—Wojkowice, 29—Sosnowiec, 30—Mysłowice).

Given the linkage distance at level 10, four groups were distinguished (Figure 2). Group I comprises similar menus only from the regular diet. In group II, there are menus from the bland diet and one menu from the regular diet (Sieradz prison). Groups III and IV comprise both regular and bland diet menus. In group III, they form three distinct subgroups: two homogeneous groups in terms of the diet type as well as one group containing four menus from the bland diet and one menu from the regular diet. In turn, group IV comprises three homogeneous subgroups: two composed of similar regular diet menus and one bland diet menu, and one subgroup containing three bland diet menus and one regular menu.

In groups III and IV, the regular diet menus are highly similar in the content of essential nutrients to the bland menus. This is the case of the regular diet menu in the Chełm prison and the bland diet in the Grądy Woniecko prison as well as the regular diet menu from Siedlce and the bland menus from the Warsaw Grochów prison. In the Sieradz prison, the content of essential nutrients in the regular diet was shown to differ only slightly from their content in the bland diet (they form one class with a linkage distance of 2.5).

## 4. Discussion

The present study shows many weaknesses of the quality of menus served in Polish prisons. This can be ascribed to the vagueness of the legal regulations imposed on the nutrition standards in these institutions, especially in terms of the nutritional value of meals. Nevertheless, the Polish recommendations are more specific than in other countries, e.g., in the Balkans [19]. The German regulations only mention that the nutritional value of prison meals should be monitored by medical staff [20]. According to the regulations in force in the Republic of Slovakia, each prisoner should receive three meals a day with a nutritional value adequate to the type and intensity of work, age, and health status. Cultural and religious requirements should also be taken into account. The break between meals should not be longer than 7 h. However, there is no information on the nutritional value [21]. The British regulations contain very broad recommendations on ensuring food safety, while there is no information on the nutritional value of meals [22].

Undoubtedly, the quality of meals in Polish prisons is determined by the low minimum catering budget allowance specified in the regulations, which makes it difficult to plan meals with adequate nutritional value. The relationship between the low financial outlays on food and the nutritional value of meals is confirmed by a report of the Supreme Audit Office on the nutrition of patients in Polish hospitals [23]. In each of the 11 hospitals where the quality of meals was audited, deficiencies in the supply of health-essential nutrients were found. This was mainly caused by the low basic catering budget allowance for food products used for preparation of meals in these hospitals, i.e., 5.97 PLN (ca. EUR 1.40). A comparison of these data with the present results shows that the food expenditure in Polish prisons was even lower, i.e., 4.22 PLN (ca. EUR 0.99) for the regular diet and 5.19 PLN (about EUR 1.22) for the bland diet. The problem of low financial outlays on food in prisons has also been reported from other European countries. According to the report of Her Majesty’s Inspectorate of Prisons, the average catering budget allowance in prisons in Great Britain is almost five times lower than that in hospitals [24].

The average energy value of the analyzed menus was 2575.5 kcal/day in the regular diet and 2569.5 kcal/day in the bland diet. These values meet the Polish recommendations for food-based energy supply for males aged 31–50 with low physical activity (2100–2600 kcal) [17], but they do not meet the recommendations for Polish prisons issued by of the Minister of Justice (> 2600 kcal) [3]. Previous investigations conducted in the Polish conditions indicated substantial discrepancies in this respect. Meals served in five prisons located in West Pomeranian Province had a calorific value of 2669.09 kcal [11], whereas one of the prisons in Warsaw served meals with a daily calorie intake of 3188.8 kcal [12]. For comparison, the average energy value of meals was 2481 kcal [25] and 2291 kcal [26] in some US prisons, 2561 kcal [27] in Britain, and 2710 kcal [28] in Australia.

The validity of the recommendations of the energy value of meals served in Polish prisons is puzzling. Establishment of such standards for inmates should take into account the possibility to purchase additional food and receive food parcels from the family [2]. There is no information on the scale of purchase of food products by Polish prisoners. However, data from Australia demonstrated that, at an average energy value of 3309 kcal in food consumed in a Queensland prison, as much as 30.5% of energy was provided by products purchased by prisoners [28]. Exact estimation of the recommended energy value of meals may be difficult due to the varied level of physical activity in Polish prisoners. Results of a study carried out in 16 Polish prisons indicate that the level of physical activity is low, moderate, and high in 45.4%, 35.3%, and 19.3% of inmates, respectively [29]. The excessive body weight exhibited by prisoners also suggests the need for reduction of the energy value of meals served in prisons. Kosendiak et al. [30] demonstrated that 57% of inmates in one of the Polish prisons had excessive body weight. Similar data were obtained in the Warsaw Detention Center, where 43.4% of prisoners had excessive body weight [12]. Based on the results of 11 studies published in 2012–2015, Agyapong et al. [31] indicated the problem of overweight and obesity in 35.6% and 23.3% of prisoners, respectively. However, prisoners are less frequently overweight than the general population [32,33]. In the case of adolescents, the largest weight gain is observed in the first weeks and months of imprisonment. There is a positive correlation between weight gain and mood disorders, which may indicate that adolescent inmates tend to exhibit emotional overeating [34].

The analysis of the menus did not reveal alarming data regarding the supply of macronutrients in the diet. Sucrose covered 8.0% of total energy intake in the case of the regular diet and 7.4% in the case of the bland diet. The World Health Organization recommends reducing the intake of free sugars to less than 10% of total energy intake [35]. The share of energy derived from saturated fatty acids was below the recommended 10% [36]. However, significant deviations from the recommendations were found in the supply of some micronutrients.

The supply of minerals was particularly high in the case of sodium, manganese, copper, and phosphorus. The problem of dietary sodium supply significantly exceeding nutritional recommendations has been reported not only in prisons [25,26,28,37] but also in kindergartens [38,39], schools [40,41], and hospitals [42]. One of the effects of excessive consumption of this mineral is the increased risk of hypertension [43]. As demonstrated by our observations, most of the sodium in the analyzed menus was added with salt during cooking. Most frequently, 5 g and in some prisons even 11 g of salt were added per day. Therefore, Polish prison workers need education in the field of the principles of proper nutrition. A good solution would be to make recommendations for the supply of sodium and other nutrients for meal planners. An insufficient level was reported in the case of calcium. The meals in both types of diets did not cover even half of the recommended intake of this nutrient. Milk and dairy products have been sporadically served in the analyzed menus. A very low calcium intake (137 mg) associated with the lack of dairy products in prison meals has also been indicated by Gould et al. [44]. Similarly, an insufficient supply of this mineral has been reported by Cook et al. [25]. The dietary calcium intake assessed in other studies of prisoner nutrition was found to meet the recommendations [28,37]. Considering the high phosphorus content, the analyzed menus were characterized by a highly unfavorable Ca:P ratio, i.e., approximately 0.30:1. This may result in arterial calcification and bone loss [45]. It is believed that the correct ratio of these two minerals should be approximately 1.5:1 [46]. As suggested in recent scientific reports, high dietary copper intake, as also shown in our study, may increase the risk of mortality from cardiovascular diseases [47] and development of type II diabetes [48]. The consumption of manganese demonstrated in the present study (6.5 mg in the regular diet and 5.9 mg in the bland diet) is over 2-fold higher than the value reported by Tripathi et al. [49]. Investigations conducted in Korea indicate cereal products (especially rice), vegetables, seasonings, fruits, and pulses as the main sources of this dietary ingredient [50].

The analysis of the vitamin content revealed an especially high supply of vitamins A, B_6_, and B_12_. In contrast, the consumption of vitamins D, C, and folate did not meet the recommended intake. An inadequate supply of these nutrients has also been observed in other Polish studies. Kucharska et al. [11] showed an excessive supply of vitamins A, B_6_, and B_12_ and deficient levels of vitamins D and C. Similarly, another study demonstrated an excessive level of vitamins A and B_12_ and insufficient content of vitamins D, C, and folate in prison meals [12]. For comparison, in some prisons in Kenya, the vitamin A intake is insufficient due to the high proportion of carbohydrates in the daily food ration and the low content of carotenoids and retinol of animal origin [51]. Prisoners may suffer from vitamin D deficiency since they spend a limited amount of time outdoors. As demonstrated by a US study, only 31% of prisoners have normal levels of this component in their blood [52]. Other authors emphasize the fact that, even when imprisoned in places with sufficient sunlight, inmates may exhibit deficiencies of this nutrient, and the problem may affect up to 90% of convicts [53]. The vitamin D supply in both diets analyzed in the present study covered only 34% of the recommendations, probably due to the low consumption of fish and fish products, eggs, and fortified foods. Therefore, it is advisable to introduce supplementation with this vitamin in Polish prisons. Similarly, the supply of vitamin C and folate was lower than the recommended intake, despite the recommendations for a minimum supply of 300 g of fruit and vegetables per day, not including potatoes. Probably due to the limited financial resources, the meals were only based on carrots, parsley, celery, beetroot, cabbage, onions, and apples. An insufficient level of vitamin C in prison meals is not often observed. Extremely low intakes (1.60 mg daily) were only reported in studies carried out in Kenya [54]. 

### Limitations

It should be emphasized that the present results are based solely on analysis of the menus, but they do not include food that prisoners can buy and receive from families. Another limitation in the results is the lack of knowledge of whether all prisoners consume the same portions of meals. This is related to the complicated prison hierarchy and confinement in cells. Therefore, further research is required to expand the knowledge of prisoner nutrition and its impact on health.

## 5. Conclusions

The results of the present study indicate that the nutrition provided in Polish prisons and detention centers does not cover the reference standards of nutrients intake. In both types of the analyzed diets, inadequate levels of vitamins (A, B_6_, B_12_, C, D, folate) and minerals (calcium, sodium, phosphorus, copper, manganese) were shown. Undoubtedly, the results of the present study indicate the need to develop new nutrition standards in Polish prisons that would propose more specific recommendations than the current standards. However, improvement of the quality of nutrition may be difficult due to the low financial outlays allocated to the purchase of food.

## Figures and Tables

**Table 1:** Energy and macronutrients provided in prisons (*n* = 30) menus per person per day and the age-specific dietary reference intake (DRI).

Observed Component	Recommended	Regular Diet		Bland Diet	
		Mean ± SD	% of DRI	Mean ± SD	% of DRI
Energy (kcal)	2100–2600 (EER)	2575.5 ± 144.9	N.A.	2569.5 ± 204.4	N.A.
Protein (g)	50–77 (RDA)	87.3 ± 6.8	N.A.	94.4 ± 8.7	N.A.
Isoleucine (mg)	N.A.	4014.7 ± 325.3	N.A.	4443.8 ± 423.6	N.A.
Leucine (mg)	N.A.	6287.9 ± 522.4	N.A.	6793.6 ± 663.6	N.A.
Lysine (mg)	N.A.	4999.2 ± 504.6	N.A.	5549.0 ± 611.1	N.A.
Methionine (mg)	N.A.	1902.2 ± 156.9	N.A.	2154.0 ± 206.3	N.A.
Cysteine (mg)	N.A.	1515.6 ± 146.8	N.A.	1618.1 ± 147.7	N.A.
Phenylalanine (mg)	N.A.	3910.2 ± 297.2	N.A.	4092.4 ± 362.4	N.A.
Tryptophan (mg)	N.A.	1068.3 ± 88.3	N.A.	1173.9 ± 114.5	N.A.
Protein (% of energy)	10–20	13.6 ± 0.8	N.A.	14.7 ± 1.1	N.A.
Fat (g)	70–87 ^1^	85.5 ± 9.9	N.A.	81.3 ± 11.8	N.A.
SFA (g)	max. 17.6–21.1	23.9 ± 3.7	N.A.	22.5 ± 4.7	N.A.
MUFA (g)	N.A.	36.9 ± 4.7	N.A.	34.2 ± 6.2	N.A.
LA (% of energy)	4	5.6 ± 1.2	140	5.8 ± 1.0	145
ALA(% of energy)	0.5	0.8 ± 0.2	160	0.8 ± 0.2	160
PUFA (g)	N.A.	19.0 ± 3.4	N.A.	19.0 ± 3.1	N.A.
Cholesterol (mg)	N.A.	234.7 ± 61.3	N.A.	197.0 ± 37.2	N.A.
Fat (% of energy)	20–35	29.3 ± 2.9	N.A.	28.1 ± 3.3	N.A.
Carbohydrates (g)	130 (RDA)	392.4 ± 29.2	N.A.	389.2 ± 35.8	N.A.
Sucrose (g)	N.A.	51.3 ± 14.1	N.A.	47.6 ± 16.3	N.A.
Fiber (g)	25 (AI)	31.6 ± 4.3	126	27.7 ± 3.4	111
Carbohydrates (% of energy)	45–65	57.1 ± 3.0	N.A.	57.1 ± 3.4	N.A.

^1^ 30% of energy from fats; EER, estimated energy requirement; SFA, saturated fatty acids; MUFA, monounsaturated fatty acids; LA, linoleic acid; ALA, alpha-linolenic acid; PUFA, polyunsaturated fatty acids; N.A., not available.

**Table 2:** Minerals provided in prisons’ (*n* = 30) menus per person per day and the age-specific dietary reference intake (DRI).

Observed Component	Recommended	Regular Diet		Bland Diet	
		Mean ± SD	% of DRI	Mean ± SD	% of DRI
Sodium (mg)	1500 (AI)	8048.3 ± 1848.2	537	4658.6 ± 1343.1	311
Potassium (mg)	3500 (AI)	4096.0 ± 475.8	117	4083.7 ± 489.6	117
Calcium (mg)	1000 (RDA)	421.2 ± 78.5	42	391.1 ± 96.9	39
Phosphorus (mg)	700 (RDA)	1360.9 ± 148.2	194	1347.1 ± 143.4	192
Magnesium (mg)	420 (RDA)	392.8 ± 53.3	94	337.4 ± 33.9	80
Iron (mg)	10 (RDA)	16.4 ± 2.2	164	13.6 ± 1.7	136
Zinc (mg)	11 (RDA)	12.9 ± 1.3	117	11.8 ± 1.1	107
Copper (mg)	0.9 (RDA)	1.7 ± 0.2	189	1.5 ± 1.0	167
Manganese (mg)	2.3 (AI)	6.5 ± 1.5	283	5.9 ± 1.2	257

**Table 3:** Vitamins provided in prisons (*n* = 30) menus per person per day and the age-specific dietary reference intake (DRI).

Observed Component	Recommended	Regular Diet		Bland Diet	
		Mean ± SD	% of DRI	Mean ± SD	% of DRI
Vitamin A (µg)	900 (RDA)	2222.3 ± 1127.0	247	2028.0 ± 521.5	225
Retinol (µg)	N.A.	1196.5 ± 1129.4	N.A.	428.7 ± 173.8	N.A.
Vitamin D (µg)	15 (AI)	5.1 ± 4.0	34	5.1 ± 5.6	34
Vitamin E (mg)	10 (AI)	14.2 ± 2.9	142	15.1 ± 3.1	151
Vitamin B_1_ (mg)	1.3 (RDA)	1.7 ± 1.2	131	1.7 ± 1.7	131
Vitamin B_2_ (mg)	1.3 (RDA)	1.6 ± 0.8	123	1.5 ± 1.0	115
Niacin (mg)	16 (RDA)	20.3 ± 2.3	127	22.5 ± 3.3	141
Vitamin B_6_ (mg)	1.3 (RDA)	2.4 ± 0.5	185	2.4 ± 0.6	185
Vitamin B_12_ (µg)	2.4 (RDA)	5.5 ± 3.8	229	2.0 ± 0.4	83
Vitamin C (mg)	90 (RDA)	57.5 ± 11.3	64	51.9 ± 14.0	58
Folate (mg)	400 (RDA)	223.5 ± 31.6	56	216.2 ± 19.0	54

**Table 4:** Lower and upper endpoints of a 95% confidence interval for the difference between mean values of regular and bland diet (${\bar{x}}_{r}$ —mean value for regular diet, ${\bar{x}}_{b}$ —mean value for bland diet).

Observed Component	${\bar{x}}_{r-}{\bar{x}}_{b}$	Lower Endpoint	Upper Endpoint
Protein (g)	−7.14	−9.96	−4.32
Fat (g)	4.27	0.34	8.19
Isoleucine (mg)	−429.05	−565.60	−292.49
Leucine (mg)	−505.73	−721.65	−289.82
Lysine (mg)	−549.86	−752.47	−347.25
Methionine (mg)	−251.77	−318.04	−185.51
Cysteine (mg)	−102.54	−155.79	−49.30
Phenylalanine (mg)	−182.23	−302.04	−62.42
Tryptophan (mg)	−105.60	−142.58	−68.62
Sodium (mg)	3389.71	2805.64	3973.78
Magnesium (mg)	55.34	39.18	71.49
Iron (mg)	2.84	2.11	3.56
Zinc (mg)	1.11	0.68	1.54
Copper (mg)	0.20	0.14	0.27
Manganese (mg)	0.64	0.14	1.14
Retinol (µg)	767.77	475.64	1059.91
Niacin (mg)	−2.23	−3.27	−1.19
Vitamin C (mg)	5.53	0.93	10.12
Vitamin B_12_ (µg)	3.55	2.58	4.52
Fiber (g)	3.93	2.52	5.35
Cholesterol (mg)	37.68	19.36	56.00
MUFA (g)	2.71	0.71	4.71
Protein (% of energy)	−1.12	−1.47	−0.78
Fat (% of energy)	1.19	0.05	2.33
